# Toll-like receptor-mediated innate immune responses by recognition of the recombinant dormancy-associated *Mycobacterium tuberculosis* proteins Rv2659c and Rv1738

**DOI:** 10.1371/journal.pone.0273517

**Published:** 2022-09-01

**Authors:** Chutiphon Saelee, Jariya Hanthamrongwit, Phyu Thwe Soe, Prasong Khaenam, Naharuthai Inthasin, Pattama Ekpo, Patchanee Chootong, Chaniya Leepiyasakulchai

**Affiliations:** 1 Faculty of Medical Technology, Department of Clinical Microbiology and Applied Technology, Mahidol University, Bangkok, Thailand; 2 Department of Medical Laboratory Technology, University of Medical Technology, Mandalay, Myanmar; 3 Faculty of Medical Technology, Center of Standardization and Product Validation, Mahidol University, Bangkok, Thailand; 4 Faculty of Medicine Siriraj Hospital, Department of Immunology, Mahidol University, Bangkok, Thailand; Rutgers Biomedical and Health Sciences, UNITED STATES

## Abstract

Tuberculosis (TB) caused by *Mycobacterium tuberculosis* (Mtb) poses a major threat to the global public health. Importantly, latent tuberculosis infection (LTBI) still impedes the elimination of TB incidence since it has a substantial risk to develop active disease. A multi-stage subunit vaccine comprising active and latency antigens of Mtb has been raised as the promising vaccine to trigger immune protection against all stages of TB. Therefore, the discovery of new antigens that could trigger broad immune response is essential. While current development of TB vaccine mainly focuses on protective immunity mediated by adaptive immune response, the knowledge on triggering the innate immune response by antigens is still limited. We showed that recombinant dormancy-associated Mtb proteins Rv2659c and Rv1738 were recognized by human innate immune recognition molecules, Toll-like receptors (TLRs) 2 and 4 by using HEK-Blue™ hTLR2/hTLR4 systems. We further demonstrated that these two proteins activated phosphorylated NF-κB p65 (Ser536) in the human CD14^+^ blood cells. We also investigated that these two proteins significantly induced level of pro- and anti-inflammatory cytokines (IL-1β, IL-6, IL-8, IL-10 and TNF-α) which were mediated through TLR2 and TLR4 pathways in human peripheral blood mononuclear cells (hPBMCs). These findings suggest that proteins Rv2659c and Rv1738 stimulated innate immune response targeting TLR2 and TLR4 to produce inflammatory cytokines, and their benefits would be valuable for the development of an effective prophylactic tuberculosis vaccine.

## Introduction

The long-term incidence of tuberculosis (TB) caused by *Mycobacterium tuberculosis* (Mtb) constitutes a major threat to public health worldwide [1]. Importantly, a quarter of the global population harbors latent infection, an anticipated reservoir of active disease [1]. Approximately 5–10% of latently-infected individuals develop active TB in their lifetime [2]. Vaccine is one of the potential strategies to combat this infectious stage [3]. However, a single Bacillus-Calmette-Guérin (BCG) since 1921 provides inadequate immune protection to all TB stages [4]. To fulfil this gap, a multi-stage subunit vaccine consisting of active and dormancy phase antigens has been proposed based on the idea of inducing the broad immune response against all forms of TB infection [5]. The theoretical conception of employing the dormancy antigens in multi-stage vaccine is to induce the immune protection against TB reactivation in LTBI group by priming the immune response from these dormancy antigens [6]. For example, a candidate vaccine (H56/IC31), consisting of two early secretory antigens of Mtb (Ag85B and ESAT-6) and a dormancy antigen (Rv2660c), increases bacterial containment and reduces disease progression in immunized mice [7]. Although most vaccines’ efficacy appears promising in pre-clinical studies, those are still pending in the clinical phase [8,9]. This is due to the lack of the suitable antigens that could elicit the protective immune compartments. Thus, identification and characterization of new antigens are crucial for multi-stage subunit vaccine development.

Mtb is well classified as the intracellular bacteria that induce the pulmonary granulomas, the immune-related structures responding to the bacterial restriction with toxic microenvironment [10]. During its quiescent stage, the dormancy-associated proteins have the mechanistic functions to help Mtb to persist by minimizing its metabolic activities and recovering from anaerobiosis [10]. To this end, the latency 48-gene complex in a two-component system (DosS and DosT) of signal transduction called dormancy survival regulon genes (DosR regulon) is the key genetic group for this response [11].

Most dormancy-associated proteins have been characterized their immune induction of adaptive immune response [12–14]. However, overall immune response controlled by targeting the innate immune receptor such as Toll-like receptors (TLRs) is now the promising strategy for subunit vaccine development [15]. TLR-mediated effects that are relevant to adjuvanticity can enhance the immune stimulation of subunit vaccine [8]. For example, synergy of a tuberculosis subunit antigen (ID93) with the TLR4 ligand GLA-SE and TLR9 ligand CpG increases the magnitude of T helper 1 (Th1) responses and protection in a mouse model [16]. Immunomodulation stimulated by TLRs can fulfil the gap of BCG vaccine that induces the short duration of memory T cells by enhancing several immunological mechanisms such as antigen presenting cell (APC) maturation, migration to infection sites, and antigen presentation [17,18]. Moreover, TLR-mediated cytokines also initiate and control adaptive immune responses [17]. For instance, murine memory T cell responses are enhanced by co-administration of cytokines (IL-1, IL-6 and TNF-α) with a Mtb-infected macrophage vaccine [19]. TLR2 enhances the stimulation of p19 (subunit of IL-23) in Th17-mediated responses against Mtb [20]. Secretion of IL-8 is responsible for CD3^+^, CD4^+^ and CD8^+^ T cell recruitment in responses to Mtb infection [21]. TLR-mediated IL-12 may increase Th1 polarization, and so IFNγ-producing CD4^+^ T cells, leading to TB protection [22]. Hence, TLR-mediated pro-inflammatory cytokines enhance the overall magnitude of the adaptive immune response.

Rv2659c (functioning in phage particle production) and Rv1738 (functioning in the shutdown of ribosomal protein synthesis) have been characterized their ability to induce adaptive immune response [23,24]. Our group utilized peptide microarray technique to gauge the specific immunoglobulins against 53 dormancy-associated Mtb proteins. Interestingly, the peptides of Rv2659c and Rv1738 were predominantly recognized by IgA in the sera of latent tuberculosis individuals (LTBIs) (unpublished data). Later we found that the number of specific recombinant protein Rv2659c IgA memory B cells (MBCs) presented in LTBIs is higher than in those with active tuberculosis [25]. Others showed that protein Rv2659c exerts its ability to escalate frequency of CD4^+^ T cells and co-expression of cytokines (IFN-γ, TNF-α and IL-2) in LTBI [26]. In addition, protein Rv1738 also induces higher levels of specific antibody in latent TB individuals compared to healthy controls [27] as well as higher IFN-γ responses specific to protein Rv1738 was found in a group of close contacts of TB patients [28]. However, the potential of dormancy-associated antigens in triggering the innate immune response has not been characterized. This is the first report illustrating the ability of proteins Rv2659c and Rv1738 in inducing innate immune responses via TLR2-TLR4 recognition. Both proteins exhibited downstream signaling induction and TLR2/TLR4-mediated, inflammatory cytokines production. Taken together, evidence of TLR-mediated innate immune response by these two dormancy-associated proteins will be beneficial in the design of multi-stage subunit tuberculosis vaccines.

## Materials and methods

### Molecular cloning and protein expression

Recombinant dormancy-associated *Mycobacterium tuberculosis* proteins Rv2659c and Rv1738 were expressed using the same protocol as described previously [25]. In brief, genomics of Rv2659c (1,128 bp) and Rv1738 (285 bp) using Mtb H37Rv strain were amplified and cloned in expression plasmids (pET24b for Rv2659c and pET28a for Rv1738), then introduced into *Escherichia coli* (*E*. *coli*) BL21 (DE3) with the induction of 0.5 mM IPTG at 37°C for 3 hours. The recombinant proteins were purified by His-tag protein purification with purity was evaluated by SDS-PAGE staining and Western blot. Endotoxins were removed using a Toxineraser^TM^ endotoxin removal kit, confirmed with a Toxinsensor^TM^ chromogenic LAL endotoxin assay kit (both from Genscript, NJ, USA).

### Blood sample collection

Heparinized blood samples of 10 ml were collected from healthy individuals (n = 3) and separated for human peripheral blood mononuclear cells (hPBMCs) by Ficoll-Hypaque density gradient centrifugation using Lymphoprep™ (STEMCELL, Canada). This study was reviewed and approved by the Central Institutional Review Board, Mahidol University, Thailand (No. MU-CIRB 2021/173.2903) for experiments involving human samples. Written informed consent was acquired from all participants and all experiments were conducted according to ethical guidelines and regulations.

### Cell culture

HEK-Blue™ hTLR2 and HEK-Blue™ hTLR4 cells (Invivogen, USA) were cultured in Dulbecco’s Modified Eagle Medium (DMEM) (Gibco, Invitrogen, UK) supplemented with 10% inactivated fetal bovine serum (FBS) (Gibco Life Technologies, UK), 50 U/l penicillin (Penstrep x100; Gibco, USA), 50 μg/ml streptomycin (Penstrep x100; Gibco, USA), 100 μg/ml Normocin (Invivogen, USA) and 1xHEK-Blue™ Selection (Invivogen, USA). Cells were incubated at 37°C in 5% CO_2_ humidified atmosphere until cell confluency reached 60–80%, and then used for recognition of hTLR2/hTLR4.

Human peripheral blood mononuclear cells were suspended in Roswell Park Memorial Institute (RPMI) 1640 medium supplemented with 10% inactivated FBS (Gibco, UK), 50 U/l penicillin (Penstrep x100; Gibco, USA) and 50 μg/ml streptomycin (Penstrep x100; Gibco, USA). Cells from healthy individuals were used to determine the kinetics of phosphorylated nuclear factor-kappa B (NF-κB) activation, quantification of human inflammatory cytokines and an anti-hTLR2/hTLR4 antibody blocking study.

### Recognition of hTLR2/hTLR4

HEK-Blue™ hTLR2 and HEK-Blue™ hTLR4 cells (Invivogen, USA), which are HEK293 cell lines engineered with hTLR2/hTLR4 and SEAP (secreted embryonic alkaline phosphatase) reporter genes were used to detect the recognitions of hTLR2/hTLR4 with proteins Rv2659c and Rv1738. TLR2/TLR4 ligands can activate NF-κB and the AP-1 promoter site resulting in SEAP production in HEK-Blue™ detection medium (Invivogen, USA) and can be measured by spectrophotometer. The color of HEK-Blue™ detection medium changes from pink to purple if recognition and interaction occur.

Recombinant proteins Rv2659c and Rv1738 in various concentrations (ranged from 100 ng/ml, 200 ng/ml, 400 ng/ml, 800 ng/ml, 1.6 μg/ml, 3.2 μg/ml and 6.4 μg/ml) were added to approximately 50,000 cells of HEK-Blue™ hTLR2 and 25,000 cells of HEK-Blue™ hTLR4 according to the manufacturer’s instructions. 1 x phosphate buffer saline (PBS) and Proteinase K (Pro K) (Vivantis Technologies, Malaysia)-digested proteins were used as the negative controls; synthetic lipoprotein FSL-1 (100 ng/ml) and lipopolysaccharide (LPS) (1 μg/ml) (both from Invivogen, USA) were used as the positive controls for TLR2 and TLR4, respectively. Protein-treated cells were incubated at 37°C in 5% CO_2_ humidified atmosphere for 16 hours before measuring the SEAP production by microplate reader (Synergy HTX Multimode Reader BioTek, USA) at 630 nm. Data from triplicate experiments (each was duplicated) was used for statistical analysis.

### Kinetics of phosphorylated NF-κB activation

Human PBMCs (2.5 x 10^5^ cells) were added in 5 ml polystyrene round-bottom tubes (Falcon, Sweden) and treated with 6.4 μg/ml of the recombinant proteins Rv2659c and Rv1738. Time intervals for this experiment were varied (0, 10, 20, 30 and 45 min). After each specified time, the cells were immediately resuspended in 4% paraformaldehyde at room temperature (RT) for 15 min. After that, cells were washed with 1 x PBS and incubated on ice with cold pure methanol for 10 min to permeabilize cells. In this step, 100% methanol which was stored at -20°C was added slowly while gently swirling cells to a final concentration of 90% methanol. Then, cells were washed twice with 1 x PBS to remove methanol, then stained with conjugated Alexa Fluor^®^ 647 anti-phosphorylated NF-κB p65 (Ser536) antibody (Cell Signaling, USA) for 1 hour at RT. Cell surface staining was subsequently performed by washing cells with FACS buffer solution and incubating with conjugated APC/Cy7 anti-human CD14 antibody (BioLegend, USA) for 15 min at 4°C. Cells were washed and resuspended with FACS buffer solution for flow cytometry analysis. Mean fluorescence intensity (MFI), proportional to the intranuclear accumulation of phosphorylated NF-κB p65 (Ser536), was acquired by flow cytometry with a FACS Canto II and data from triplicate experiments were analyzed by FlowJo software (both from BD Biosciences, USA).

### Quantification of human inflammatory cytokines

Human PBMCs (5 x 10^5^ cells) were plated in 96-well cell culture plates (Corning, NY, USA) and treated with recombinant proteins Rv2659c and Rv1738 in various concentrations (400 ng/ml, 800 ng/ml, 1.6 μg/ml, 3.2 μg/ml and 6.4 μg/ml). Untreated cells were used as the negative control, while LPS (1 μg/ml) was used as the positive control. The experiments were incubated at 37°C in 5% CO_2_ humidified atmosphere for 24 hours. This 24-hour incubation was selected to cover all cytokines since most cytokine concentrations are produced adequately at this time [29,30]. Culture supernatants were collected and stored at -80°C before performing cytometric bead array (CBA) analysis for quantification of released cytokines.

CBA assay (Human Inflammatory Cytokines Kit; BD Biosciences, USA) is a multiplex analysis method using specific antibody-conjugated beads with known size and fluorescence for measuring a set of cytokines (IL-1β, IL-6, IL-8, IL-10 and TNF-α) in the culture supernatants. In brief, 50 μl of capture beads specific to cytokines, 50 μl of culture supernatant and 50 μl of PE-conjugated detection antibodies were mixed and incubated for 3 hours at RT. Then, beads were washed and resuspended with wash buffer according to the manufacturer’s instructions. The intensity of fluorescence from beads represents type of cytokine, while from PE-conjugated detection antibody is proportional to the quantity of cytokine. Samples of 3 individual subjects (each was duplicated) were measured by flow cytometry with a FACS Canto II and data were analyzed by FCAP array software (both from BD Biosciences, USA).

### Anti-hTLR2/hTLR4 antibody blockings

Human PBMCs at the density of 2.5 x 10^5^ and 5 x 10^5^ were used for hTLR2 and hTLR4 blocking groups, respectively. The hPBMCs were plated in 96-well cell culture plates (Corning, NY, USA); 5 μg/ml (final concentration) of anti-hTLR2 antibody (PAb-hTLR2; Invivogen, USA) and anti-hTLR4 antibody (PAb-hTLR4; Invivogen, USA) were added. The antibody-blocked cells were then incubated at 37°C in 5% CO_2_ humidified atmosphere for 1 hour before treating with 6.4 μg/ml of recombinant proteins Rv2659c and Rv1738. Importantly, antibody blocking groups were treated with TLR2 agonist FSL-1 (100 ng/ml) and TLR4 agonist LPS (1 μg/ml); untreated cells were also applied as the systemic control for hTLR2-hTLR4 effective blocking assessment. Then, the duplicate experiments were incubated with stated conditions for 24 hours.

Along with these antibody-blocking groups, unblocking groups were similarly treated for use in comparisons. In this step, unblocked cells were treated with 6.4 μg/ml of recombinant proteins Rv2659c and Rv1738. Untreated cells were used as the negative control, while FSL-1 (100 ng/ml) and LPS (1 μg/ml) were used as the positive controls for TLR2 and TLR4 respectively.

The cell culture supernatants from antibody blocking groups and unblocking groups were collected and stored at -80°C before performing the CBA analysis. Data from samples of 3 individual subjects (each was duplicated) was used for statistical analysis.

### Statistical analysis

One-way ANOVA with Dunnett’s multiple comparison tests were performed to compare groups of positive controls and proteins varied by concentrations with the negative control for recognition of hTLR2/hTLR4 and quantification of human inflammatory cytokines. One-way ANOVA with Sidak’s post hoc test was performed to compare antibody blocking with unblocking groups for anti-hTLR2/hTLR4 antibody blockings. Results with a p-value < 0.05 were considered as statistically significant. GraphPad Prism 8 (San Diego, CA, USA) was utilized for analysis of results and generation of graphs.

## Results

### Recombinant Rv2659c and Rv1738 proteins of *M*. *tuberculosis* are recognized by hTLR2 and hTLR4

**Fig 1:**
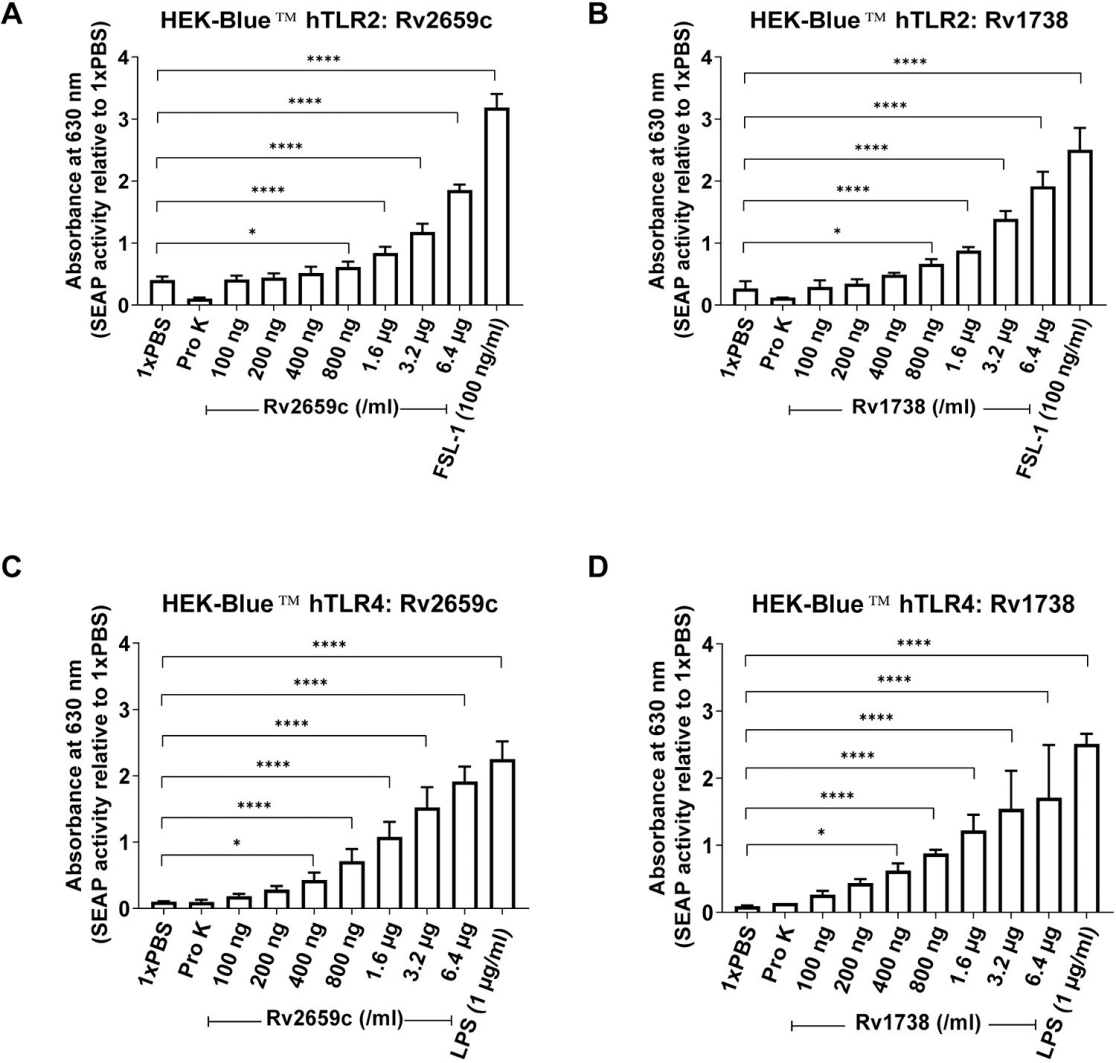
Recognition of recombinant proteins Rv2659c and Rv1738 by hTLR2 and hTLR4. HEK-Blue™ hTLR2 and HEK-Blue™ hTLR4 cells were treated with different concentrations (100 ng/ml, 200 ng/ml, 400 ng/ml, 800 ng/ml, 1.6 μg/ml, 3.2 μg/ml and 6.4 μg/ml) of proteins Rv2659c (**A** and **C**) and Rv1738 (**B** and **D**) for 16 hours before measuring SEAP production by microplate reader. 1 x PBS and Pro K-digested protein were used as the negative controls, while FSL-1 (100 ng/ml) and LPS (1 μg/ml) were served as the positive controls for TLR2 and TLR4, respectively. Data are represented as mean ± SD of triplicate experiments (Each was duplicated). One-way ANOVA with Dunnett’s multiple comparison test was used for statistical analysis. *p* values are represented with asterisk symbols (*p < 0.05 **p < 0.01, ***p < 0.001 and ****p < 0.0001).

The membrane-bound Toll-like receptors are important pattern recognition receptors (PRRs) that recognize an array of microbe-derived molecules. Mtb antigens are recognized by TLR2 and TLR4 on the surfaces of host innate immune cells [31]. For instance, dormancy-associated antigen Rv2660c interacts with TLR2 on macrophages and produces cytokines that help maintain Mtb in latency phase, while RpfB incites DCs towards Th1/Th17 cell expansion in a TLR4-dependent pathway [32,33]. However, recognition and interaction of Rv2659c and Rv1738 via TLR2/TLR4 are not yet identified. To examine whether these two dormancy-associated antigens can be recognized by TLR2/TLR4, HEK-Blue™ hTLR2 and HEK-Blue™ hTLR4 (co-transfected human embryonic kidney 293 cells that stably overexpress individual human TLR2, human TLR4 and a reporter signal NF-κB-inducible SEAP) were employed. We produced recombinant Rv2659c and Rv1738 proteins in an *E*. *coli* system free of endotoxin contamination. NF-κB reporter cells suspended in HEK-Blue™ detection medium were treated with various concentrations of these two proteins (100 ng/ml, 200 ng/ml, 400 ng/ml, 800 ng/ml, 1.6 μg/ml, 3.2 μg/ml and 6.4 μg/ml). TLR2/TLR4 activation was assessed by measuring NF-κB-dependent SEAP, reflected by colorimetric shift and higher absorbance of detection media. Upon treatment, proteins Rv2659c and Rv1738 induced significantly greater NF-κB-dependent SEAP activity in a dose-dependent manner (Fig 1). Increased absorbance from HEK-Blue™ hTLR2 and hTLR4 cells incubated with Rv2659c (initiated at 800 ng/ml and 400 ng/ml, respectively) was found in comparison with 1 x PBS-treated cells (Fig 1A and 1C). Similar results were observed with Rv1738 (Fig 1B and 1D). Moreover, TLR2 agonist FSL-1 (100 ng/ml) and TLR4 agonist LPS (1 μg/ml), serving as positive controls, induced the strongest absorbance from each human TLR reporter system. A batch of protein, digested with proteinase K, was added to the cells for exclusion of non-specific or LPS-mediated induction of SEAP. In every experiment, the absorbance from hTLR2 and hTLR4 reporter cell models treated with proteinase K-digested proteins was strongly hindered (Fig 1). These results indicated that proteins Rv2659c and Rv1738 can be recognized by hTLR2/hTLR4 and that these interactions sequentially induced NF-κB activation.

### Recombinant proteins Rv2659c and Rv1738 induce phosphorylated NF-κB activation in human CD14^+^ cells

The ubiquitous transcription factor NF-κB regulates the production of inflammatory cytokines in response to various stimuli from host innate immune cells such as macrophages, dendritic cells and neutrophils [34]. Recognition of TLR2 and TLR4 on the surface of these cells leads to canonical NF-κB activation, by inducing the phosphorylation cascade of multi-subunit IκB (IKK) kinase complex and degradation of IκBα, an inhibitor of NF-κB [34]. This mechanism contributes to the nuclear translocation of free NF-κB transcription factors to target site-specific, inflammatory-cytokine related genes [34]. Phosphorylation and nuclear translocation of NF-κB in mouse macrophages responding to inflammatory stimuli is rapidly (5 minutes) induced by the activation at site serine 536 of p65 [35]. Therefore, we examined the intranuclear accumulation of phosphorylated NF-κB p65 at residue serine 536 of human monocytes in the presence of recombinant proteins Rv2659c and Rv1738 for maximum at 45 minutes using flow cytometry.

**Fig 2:**
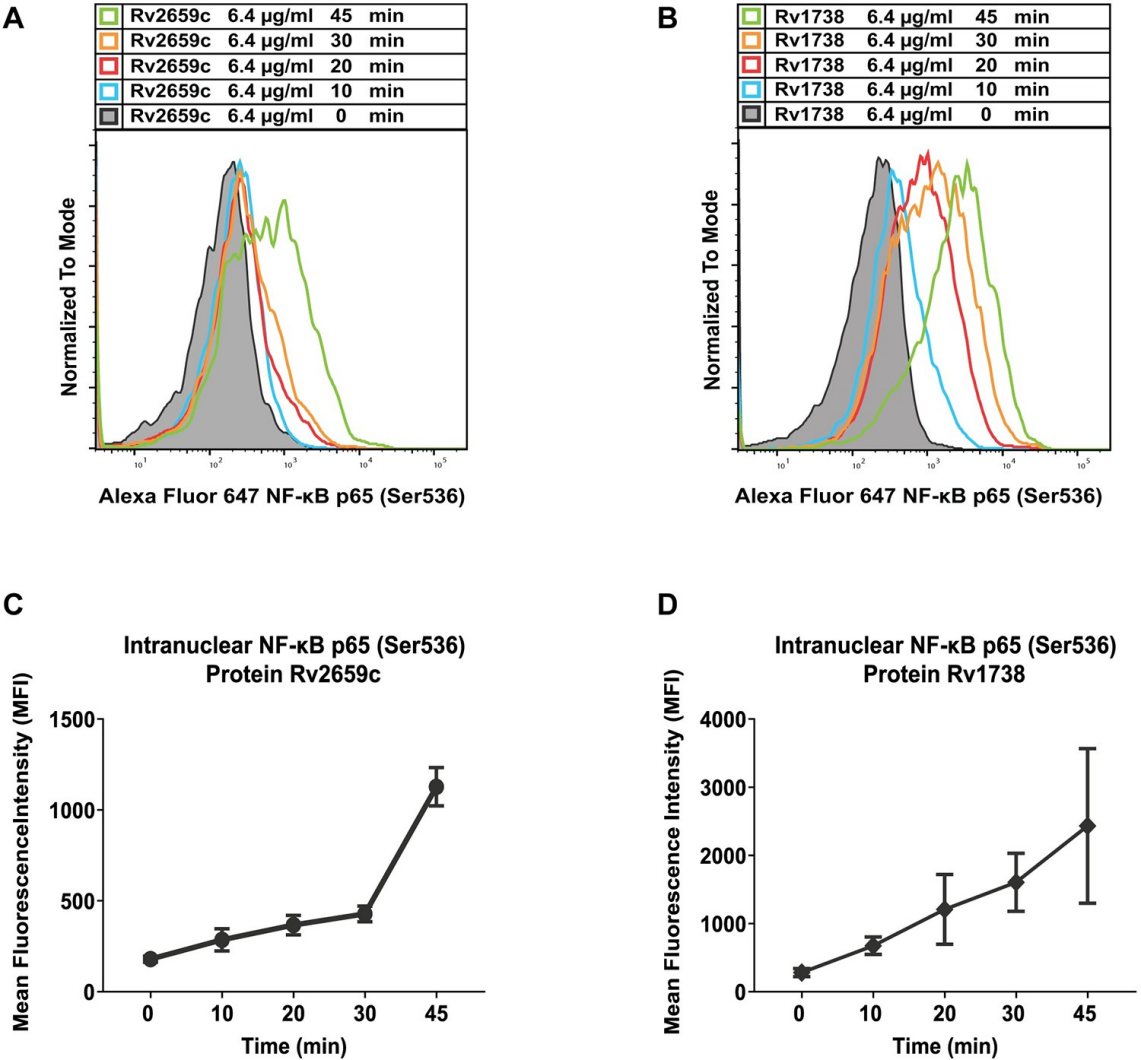
Phosphorylation of NF-κB p65 (Ser536) in human CD14^+^ population after treatment with 6.4 μg/ml of recombinant proteins Rv2659c or Rv1738. Representative flow cytometric histograms showing translocation of NF-κB p65 (Ser536) in human CD14^+^ population after treatment of hPBMCs with proteins Rv2659c (**A**) or Rv1738 (**B**). Mean fluorescence intensity (MFI) of phosphorylated NF-κB p65 (Ser536) from cells treated with proteins Rv2659c (**C**) or Rv1738 (**D**) at each timepoint. Data were acquired by flow cytometry, analyzed by FlowJo software and represented as mean ± SD of triplicate experiments.

Human PBMCs were treated with 6.4 μg/ml of recombinant proteins Rv2659c or Rv1738 at different timepoints (0, 10, 20, 30 and 45 min). A previous study demonstrated that NF-κB phosphorylation occurred within minutes [35]. At the appropriate timepoints, cells were immediately fixed, permeabilized and stained with antibodies. Intranuclear phosphorylated NF-κB in human CD14^+^ monocytes were stained with anti-phosphorylated NF-κB p65 (Ser536) antibody and mean nuclear NF-κB fluorescence intensity was acquired by flow cytometry. The kinetics of phosphorylated NF-κB translocation in human CD14^+^ monocytes is shown in Fig 2.

The results demonstrated that the overall level of phosphorylation signal increased continuously over time. In particular, recombinant protein Rv2659c activated a gradual increase of MFI during the initial 30 min after which the signal significantly escalated at 45 min (Fig 2A and 2C). In contrast, recombinant protein Rv1738 induced a more gradual upward trend of NF-κB kinetics over 45 min (Fig 2B and 2D). In this study, the mean nuclear NF-κB fluorescence intensity stimulated by recombinant protein Rv1738 was greater than that of protein Rv2659c. This reflected a difference in the immune induction abilities of the two proteins.

Thus, the recombinant proteins Rv2659c and Rv1738 induced activation of phosphorylated NF-κB p65 (Ser536) which accumulated in the nuclei of human CD14^+^ monocytes. This nuclear translocation of IκBα-unbound NF-κB was mediated by phosphorylation of the IKK kinase subunit to target specific inflammatory-cytokine genes in human CD14^+^ monocytes, producing pro-inflammatory cytokines in response to proteins Rv2659c and Rv1738.

### Recombinant proteins Rv2659c and Rv1738 induce pro- and anti-inflammatory cytokines in hPBMCs

The production of pro-inflammatory cytokines induced by canonical NF-κB activation has been extensively studied in innate immune cells such as monocytes and macrophages [34,36]. The cascades of cytokines released by these cells initially mount inflammatory responses and also recruit T lymphocytes and other cells to mount adaptive immune responses [37].

**Fig 3:**
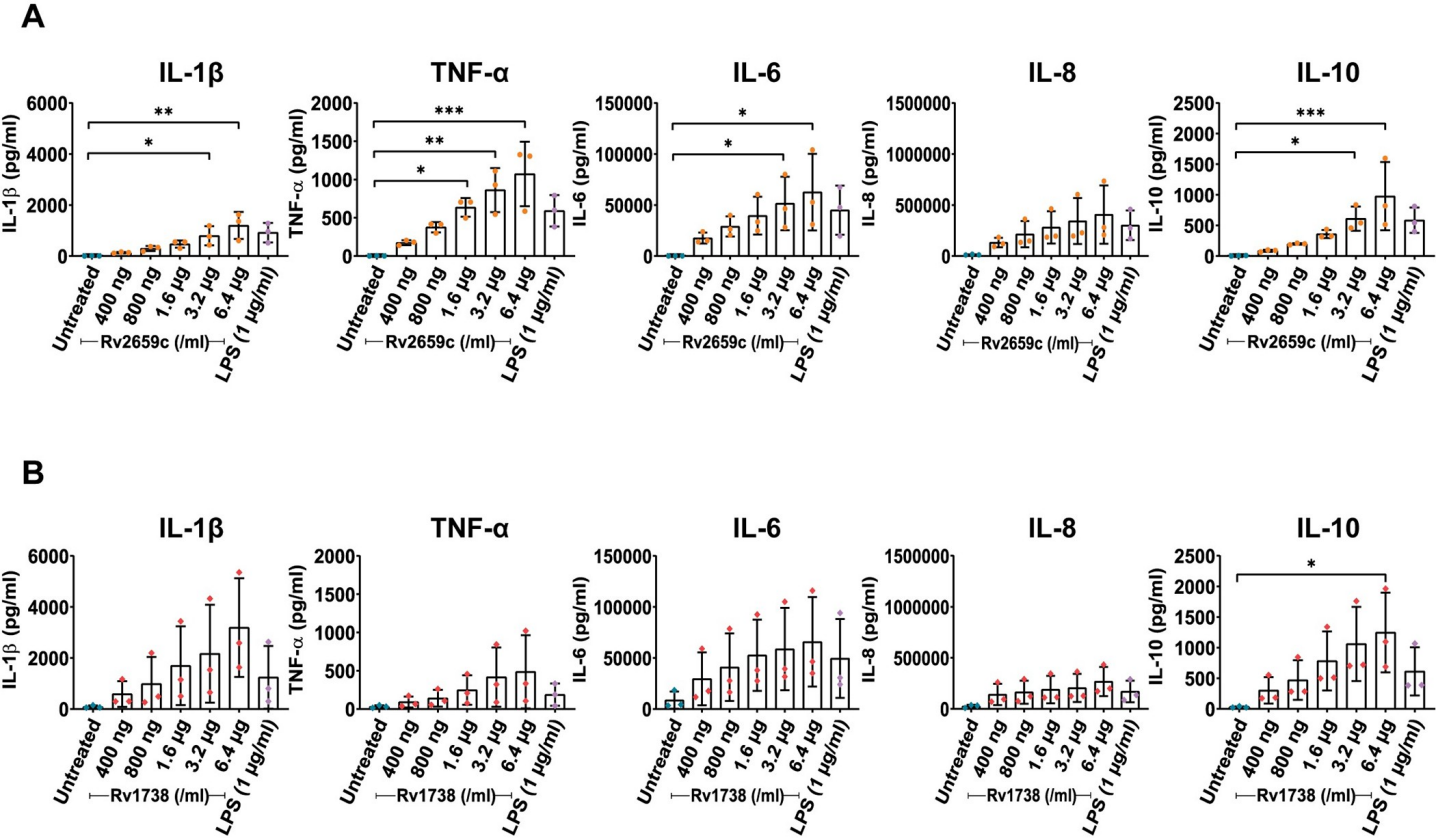
Recombinant proteins Rv2659c and Rv1738 induced a set of pro- and anti-inflammatory cytokines in hPBMCs. **(A)** Cytokine production stimulated by protein Rv2659c. **(B)** Cytokine production stimulated by protein Rv1738. Human PBMCs were treated with different concentrations of the proteins (400 ng/ml, 800 ng/ml, 1.6 μg/ml, 3.2 μg/ml and 6.4 μg/ml) for 24 hours. Untreated cells served as the negative control, while LPS (1 μg/ml) served as the positive control. CBA assay was performed to detect IL-1β, IL-6, IL-8, IL-10 and TNF-α in the culture supernatants. Data were acquired by flow cytometry, analyzed by FCAP array software and represented as mean ± SD of 3 individual subjects (each experiment was done in duplicate). One-way ANOVA with Dunnett’s multiple comparison test was performed for statistical analysis. *p* values are represented with asterisk symbols (*p < 0.05 **p < 0.01, ***p < 0.001 and ****p < 0.0001).

We next investigated the ability of recombinant proteins Rv2659c and Rv1738 through activation of human immune cells to produce and release an array of inflammatory cytokines. In this study, hPBMCs were treated with various concentrations (from 400 ng/ml to 6.4 μg/ml) of the proteins. Pro- and anti- inflammatory cytokines (IL-1β, IL-6, IL-8, TNF-α and IL-10) were quantitated in the cell culture supernatants using a cytometric bead array (CBA) assay. Concentrations of IL-1β, IL-6, IL-8, TNF-α and IL-10 were found to increase in a dose-dependent manner from cells treated with 400 ng/ml to 6.4 μg/ml of proteins Rv2659c and Rv1738 (Fig 3A and 3B). Recombinant protein Rv2659c induced more TNF-α and IL-8 than did protein Rv1738. In contrast, IL-1β and IL-10 production was greater after induction by Rv1738 than Rv2659c. The amount of IL-6 was released to similar levels from hPBMCs treated with proteins Rv2659c and Rv1738. Cells treated with LPS (1 μg/ml), a positive control, induced the strong cytokine responses whereas negative controls (unstimulated cells) produced no elevation of cytokine levels.

These results suggested that both recombinant proteins Rv2659c and Rv1738 were able to induce hPBMCs to secrete pro- and anti- inflammatory cytokines. However, the triggering of these cytokine responses may also result via other signaling pathways. For instance, rapid production of TNF-α occurs through APCs and TCR-engagement with CD8^+^ naïve T cells [38]. Also, subclasses of Scavenger receptors bound with diverse ligands can mediate NF-κB activation and production of pro-inflammatory cytokines and chemokines such as IL-1β, IL-6, TNF-α, IL-2, IL-10 [39]. Thus, we next performed experiments to prove whether the cytokine stimulation by the recombinant proteins Rv2659c and Rv1738 is via the TLR-mediated signaling pathway.

### The production of pro- and anti-inflammatory cytokines in hPBMCs were mediated through hTLR2 and hTLR4 signaling

In order to confirm that those cytokines were produced via hTLR2 and hTLR4 signaling pathways, experiments for blocking these receptors were conducted. In this study, the two receptors were blocked in hPBMCs with anti-hTLR2 and anti-hTLR4 antibodies before treating with 6.4 μg/ml of recombinant proteins Rv2659c or Rv1738. At the same time, hPBMCs treated with 6.4 μg/ml of proteins Rv2659c and Rv1738 served as the unblocking group. The concentrations of pro- and anti- inflammatory cytokines (IL-1β, IL-6, IL-8, TNF-α and IL-10) in the cell culture supernatants were measured using CBA analysis. The amounts of cytokines found in blocking and unblocking groups were compared.

**Fig 4:**
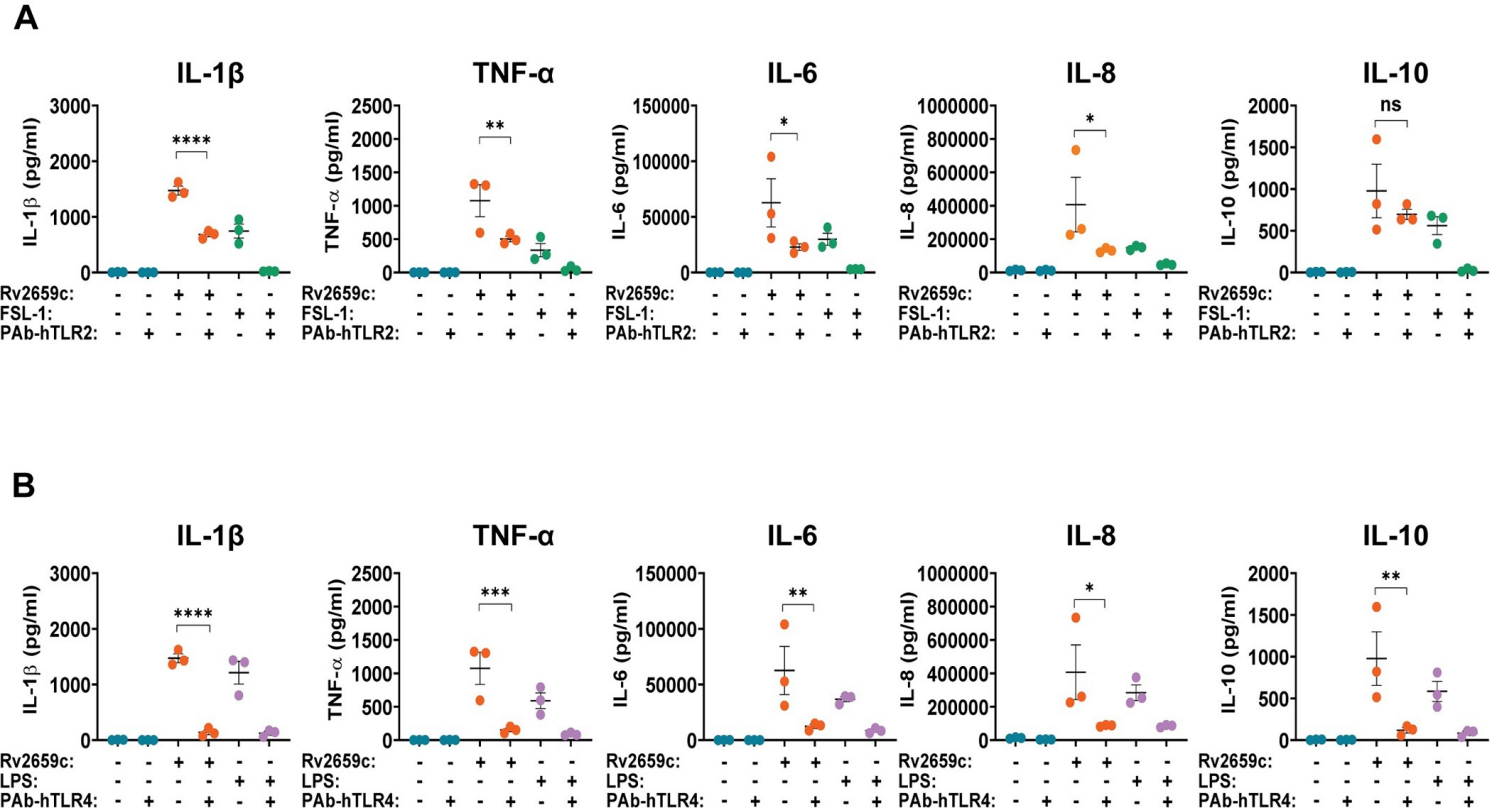
Recombinant protein Rv2659c induced the production of pro- and anti-inflammatory cytokines in hPBMCs through hTLR2 and hTLR4 interactions. **(A)** Cytokine production from hTLR2 blocking group. **(B)** Cytokine production from hTLR4 blocking group. The ligand-binding pockets of hTLR2 and hTLR4 in human PBMCs were blocked with 5 μg/ml of anti-hTLR2 and anti-hTLR4 antibodies, respectively, for 1 hour. Protein Rv2659c (6.4 μg/ml) was then added and incubated for the next 24 hours. For the blockings of untreated cells, FSL-1 (100 ng/ml) and LPS (1 μg/ml) served as systemic controls. IL-1β, IL-6, IL-8, IL-10 and TNF-α in the culture supernatants were quantitated by CBA assay. Data were evaluated by flow cytometry, analyzed by FCAP array software and represented as mean ± SEM of 3 individual subjects (each was done in duplicate). One-way ANOVA with Sidak’s post hoc test was used for statistical analysis. *p* values are represented with asterisk symbols (*p < 0.05 **p < 0.01, ***p < 0.001 and ****p < 0.0001).

The level of pro- and anti- inflammatory cytokines (IL-1β, TNF-α, IL-6 and IL-8) induced by recombinant protein Rv2659c significantly decreased 0.536, 0.533, 0.635 and 0.673-fold in anti-hTLR2 antibody-treated compared to untreated hPBMCs, respectively (Fig 4A). The level of IL-10 was the only cytokine in the hTLR2 blocking group that, though it slightly decreased, did not significantly change (Fig 4A). The level of all cytokines (IL-1β, TNF-α, IL-6, IL-8 and IL-10) stimulated by protein Rv2659c after anti-hTLR4 antibody treatment of hPBMCs were significantly declined (0.905, 0.858, 0.803, 0.787 and 0.880-fold, respectively) compared to those from untreated hPBMCs (Fig 4B). These results indicated that the production of inflammatory cytokines by hPBMCs induced by protein Rv2659c were mediated through hTLR2 and hTLR4 signaling pathways. However, the pathway of hTLR2 had only a minor influence on the level of IL-10. Interestingly, the remarkable decreases of cytokine levels in the hTLR4 blocking group were more than those in the hTLR2 blocking group. This suggested that the production of cytokines was mostly affected through the hTLR4 signaling pathway.

**Fig 5:**
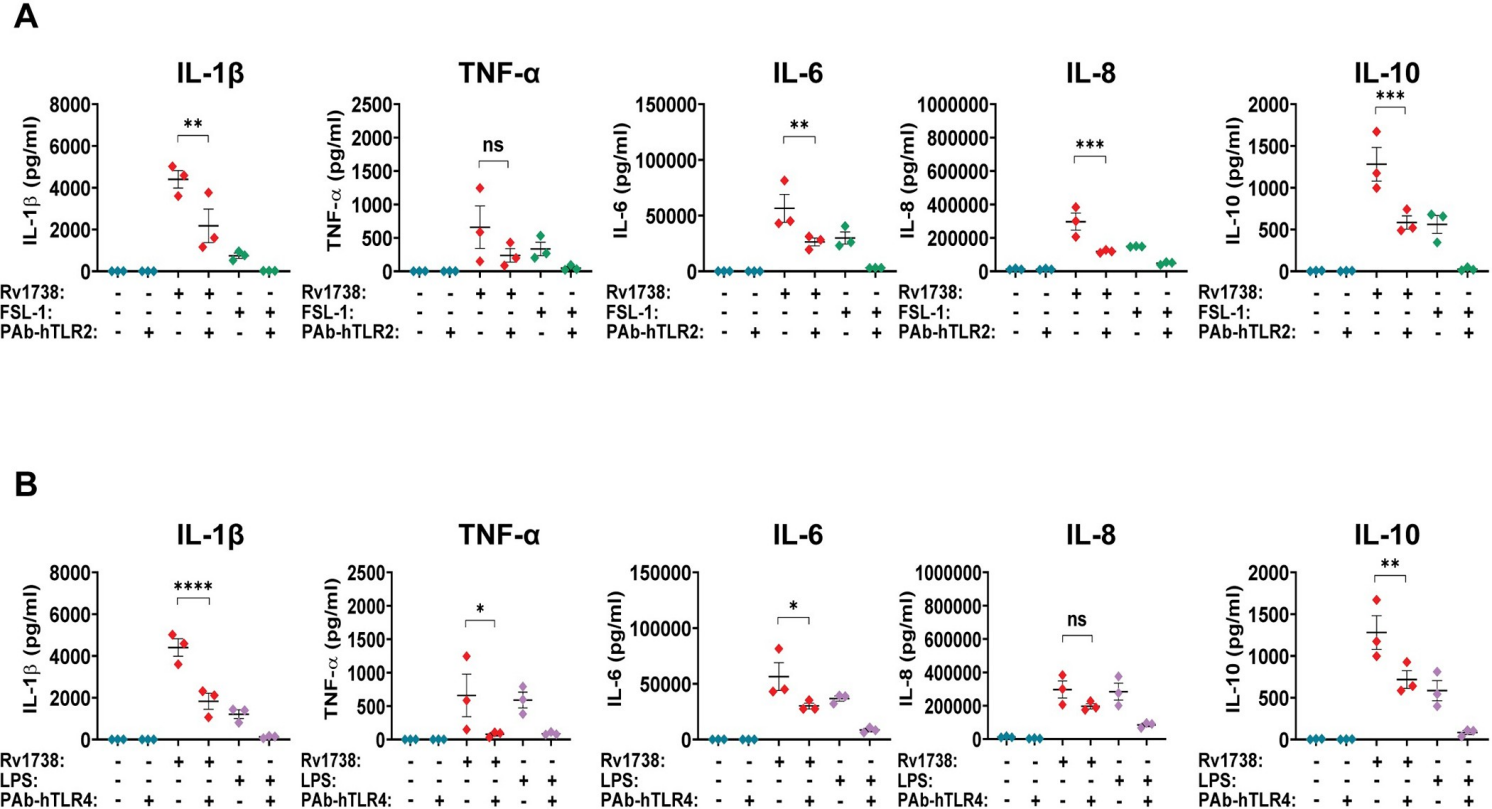
Levels of pro- and anti- inflammatory cytokines stimulated by protein Rv1738 in hPBMCs via hTLR2 and hTLR4 pathways. **(A)** Cytokine production from hTLR2 blocking group. **(B)** Cytokine production from hTLR4 blocking group. The ligand-binding pockets of hTLR2 and hTLR4 in human PBMCs were blocked with 5 μg/ml of anti-hTLR2 and anti-hTLR4 antibodies, respectively, for 1 hour. Recombinant protein Rv1738 (6.4 μg/ml) was subsequently added and incubated for the next 24 hours. Blockings of untreated cells, FSL-1 (100 ng/ml) and LPS (1 μg/ml) were applied as the systemic controls. A set of cytokines (IL-1β, IL-6, IL-8, IL-10 and TNF-α) in the culture supernatants was quantitated by CBA assay. Data were evaluated by flow cytometry, analyzed by FCAP array software and represented as mean ± SEM of 3 individual subjects (each experiment was done in duplicate). One-way ANOVA with Sidak’s post hoc test was used for statistical analysis. *p* values were represented with asterisk symbols (*p < 0.05 **p < 0.01, ***p < 0.001 and ****p < 0.0001).

In the meantime, hTLR2 blocking group of hPBMCs treated with recombinant protein Rv1738 had significant declines for all cytokine levels (except TNF-α) with the following fold changes: 0.506, 0.535, 0.598 and 0.544 for cytokines IL-1β, IL-6, IL-8 and IL-10, respectively (Fig 5A). Similarly, the concentrations of all inflammatory cytokines (except IL-8) remarkably reduced 0.584, 0.881, 0.467 and 0.440-fold for IL-1β, TNF-α, IL-6 and IL-10 in hTLR4 blocking cells stimulated by recombinant protein Rv1738, respectively (Fig 5B). Thus, these results specified that the production of pro- and anti-inflammatory cytokines induced by protein Rv1738 in hPBMCs was also mediated through hTLR2 and hTLR4 signaling pathways.

All in all, even though the production of cytokines by stimulation of hPBMCs with these two proteins may occur via various pathways, our results have proven that recombinant proteins Rv2659c and Rv1738 can activate NF-κB via the pattern recognition receptors hTLR2 and hTLR4, and result in production of inflammatory cytokines.

## Discussion

Latent tuberculosis infection poses a threat to the global control of tuberculosis due to the TB reactivation mechanism that increases overall TB incidence [1]. According to WHO, the estimated number of people who have this stage is around one-fourth of the world’s population [1]. One of recommended strategies to prevent the active TB progression in LTBI group is the vaccination [3]. However, a single approved BCG vaccine cannot induce enough immune protection against all TB stages [4]. Hence, to fulfil this gap, multi-stage subunit vaccine composing of active and latent phase antigens has been raised with the concept of stimulating the broad-immune response to both active and latent TB [5]. In this vaccine development, protein selection is crucial [5]. Nowadays, the immunodominance of active phase proteins such as ESAT6 and Ag85B has been elucidated, while new latency antigens are still required [5]. Most researchers now focus on the evaluation of new latency antigens in stimulating adaptive immune response [12–14]. Nonetheless, innate immune response is also important to enhance more effective immune response through the specialized control of adaptive immunity in the vaccination, especially multi-stage subunit vaccine [15,17,18].

Innate immune response targeting TLRs is now a prominent strategy due to its adjuvanticity [15,17,18]. Several studies of TB vaccine development elicited the power of TLR in enhancing the overall immune response [15]. For example, synergy of the ID93 fusion protein (which includes Rv1813, Rv2608, Rv3619 and Rv3620) with TLR4 agonist GLA-SE and TLR9 agonist CpG increased co-expression of IFN-γ, TNF-α, and IL-2 production in immunized mice [16]. In the concept of vaccination, triggering of long-lived, antigen specific antibody secreting cells (ASCs) and long-lasting T cell response is crucial to provide adequate immune protection [18]. Specifically, compartment related to TB protection is the adaptive T cell response [40]. However, transient expression of central memory T cells was induced by BCG vaccine, while those T cell populations stimulated by subunit vaccine expressed for 2 years stated in the comparative study [5]. To fulfil this gap of BCG vaccine, immunological mechanisms of TLRs that contributed to adjuvant activities has been proposed to increase clonal expansion of T cells by enhancing the strength and duration of peptide-MHC and TCR complexes [18]. Moreover, effects of TLR-mediated cytokine response are intended to improve the maintenance and reduce the clonal contraction of T cells [18]. These strategies might eventually improve the duration of TB protection since the vaccine efficacy is lower according to the increase of ages.

Among TLR members, TLR2 and TLR4 are the major receptors involved in the recognition and interaction of Mtb proteins, especially those expressed on the surface of alveolar macrophages [31,41]. Several types of evidence indicate the protective role played by these receptors against Mtb [31]. For example, TLR2-dependent autophagy and apoptosis is stimulated by LpqH of Mtb and restrains the growth of Mtb in macrophages [42]. Defective granuloma and reduced bacterial clearance are observed in TLR2-deficient mice [43]. TLR2-engagement is also important to initiate Mtb-specific T cell polarization to Th1 and specific memory T cell expansion [44,45]. Besides the TLR2 findings, escalation in bacterial burden and necrotic lung inflammation develop in TLR4-knockout mice [46]. Enhancement of memory CD4^+^ and CD8^+^ T cells in response to reduced bacterial lung burdens is also mediated through TLR4 [47]. TLR4-NOX2-facilitated Mtb killing occurs through ROS production and phagocytosis augmentation [48]. Hence, TLR2 and TLR4 agonists can both be included in further Mtb vaccine designs.

In this study, proteins Rv2659c and Rv1738 were proposed to elucidate their innate immune induction ability since a role in adaptive immunity was revealed previously [25–28]. We demonstrated that proteins Rv2659c and Rv1738 were recognized by hTLR2 and hTLR4 using HEK-Blue engineered cell lines. This recognition subsequently contributed to TLR-mediated activation of phosphorylated NF-κB p65 (Ser536) to translocate sequestered cytosolic NF-κB into the nuclei of human CD14^+^ monocytes. The NF-κB activation within minutes is valid evidence that these proteins can trigger the robust TLR-mediated innate immune signaling, specifically, canonical pathway [34]. Evaluation of NF-κB activation by intranuclear staining is successfully employed without the nuclear protein extraction [35]. It is consistent to the results of this study that this technique can detect the increase pattern of NF-κB activation. In addition, different patterns of phosphorylated NF-κB activation might have been associated to each protein, since Rv1738 had greater shift of MFI than did Rv2659c. These findings were in agreement with previous studies that IL-1β and sCD40L conferred different patterns of NF-κB activation during a 30-minute exposure [49]. Varied concentrations of stimuli, dynamics of NF-κB activation state and cellular heterogeneity are also important factors in evaluating phosphorylated NF-κB [50].

Besides NF-κB activation, cytokines also reflected the ability of triggering innate immunity through the mediation of TLR pathways. Secretion of pro- and anti-inflammatory cytokines (IL-1β, IL-6, IL-8, IL-10 and TNF-α) after activation by DNA-bound NF-κB was exhibited in both Rv2659c- and Rv1738-treated hPBMCs. Secretion of most cytokines induced by protein Rv2659c utilized the TLR4 more than the TLR2 pathway (dependent mediation which was still significant except with IL-10). Overall, TLR2 and TLR4 equivalently shaped cytokine production in Rv1738-treated cells with the exception that TLR2 and TLR4 that played a greater role for TNF-α and IL-8 respectively. Inconsistent cytokine patterns might have been due to variation of individual samples or differing degrees of cytokine release with the two proteins, thus necessitating further research. In this study, we performed protein stimulation experiment only one timepoint at 24-hr incubation to assure that the cytokine concentrations are sufficient to detect. According to the previous studies, cytokine levels of IL-1β, IL-10 and IL-6 are higher for 24-hour incubation than 4-hour incubation in LPS-stimulated hPBMCs [29]. Concentrations of cytokines IL-1β, IL-6, IL-8, IL-10 are also greater at 24-hour incubation than those of 4, 6 and 8-hour incubation in LPS-treated hPBMCs [30]. Importantly, the functional roles of these TLR2-TLR4 mediated cytokines in TB protection has been described [51]. For instance, TNF-α enables granuloma maintenance, activation of phagocytes and control of chronic TB infection [52]. Deterioration of IFN-γ responses and depletion of CD4^+^ and CD8^+^ T cells, examined with TNF-α blockers, suggest its function in adaptive immunity [53]. IL-1β receptor-knockout mice exhibit more susceptibility to TB infection and have increased mycobacterial growth in their lungs [54]. IL-1β is also crucial for Th17 responses in TB [55]. Secretion of IL-8 also exploited the ability in T cell recruitment and Mtb phagocytosis by tissue monocytes and macrophages [21]. However, concerning point for IL-6 application emerged when there were positive and negative effects [56]. IL-6 is associated with increased disease severity by suppressing the IFN-γ responses of uninfected macrophages [57]. In contrast, IL-6 knockout mice have an early increase of bacterial load associated with delayed IFN-γ responses [58].

Due to the aim of this study is to elucidate the ability of proteins in triggering TLR-mediated innate immune response for the application as adjuvants in multi-stage subunit vaccine, protein assessments on TLR-mediated recognition and cytokine response are adequate to reflect the adjuvanticity characteristics [59]. We rather concern that how adjuvanticity of proteins Rv2659c and Rv1738 compared to other available proteins that elicited the power of adjuvant activities. The insight knowledge of proteins Rv2659c and Rv1738 in triggering TLR-mediated innate immune response could be used as an alternative protein selection together with the active phase proteins to develop a multi-stage subunit vaccine. This vaccine could be further tested its safety, immunogenicity and immune protection in animal models [60]. The critical point to utilize these 2 proteins as the adjuvants is to determine the optimal concentrations.

In conclusion, recombinant dormancy-associated Mtb proteins Rv2659c and Rv1738 interact with hTLR2/hTLR4 inducing inflammatory cytokine responses. These cytokines might be involved in T cell activation and differentiation, maintenance of Mtb-restricted granulomas and inhibition of TB reactivation. Our results suggest that the proteins have a promising functional role in immune responses, and would be beneficial in antigen selection for future multi-stage *M*. *tuberculosis* subunit vaccine development.

## Supporting information

S1 Dataset (XLSX)
